# Network analysis dataset of system dynamics models

**DOI:** 10.1016/j.dib.2019.104723

**Published:** 2019-10-28

**Authors:** Gergely Honti, Gyula Dörgő, János Abonyi

**Affiliations:** MTA-PE Complex Systems Monitoring Research Group, University of Pannonia, Veszprem, Hungary

**Keywords:** Systems dynamics models, Model analysis, Network analysis, Sustainability, Cause-effect analysis

## Abstract

This paper presents a tool developed for the analysis of networks extracted from system dynamics models. The developed tool and the collected models were used and analyzed in the research paper, Review and structural analysis of system dynamics models in sustainability science [1]. The models developed in Vensim, Stella, and InsightMaker are converted into networks of state-variables, flows, and parameters by the developed Python program that also performs model reduction, modularity analysis and calculates the structural properties of the models and its main variables. The dataset covers the results of the analysis of nine models in sustainability science used for policy testing, prediction and simulation.

**Table:** Specifications Table

Subject	Modelling and Simulation
Subject area	network theory, sustainable development, sustainability, systems dynamics, model analysis
More specific subject area	System Dynamics, network analysis, model comparison
Type of data	Network data, Models of sustainability science, Python Code
How data was acquired	Systematic literature overview of the system dynamics models. The automated analysis was carried out by the developed Python-based analysis tool also available on the repository.
Data format	**Raw and analyzed data** Raw data: models are available on the repository Analyzed data: the full analysis is part of the research article.
Experimental factors	The model collection is a result of the systematic literature overview of the past five years (2013-early 2019) in the topic of sustainability. All 130 models are listed and described in the appendix section of the research article. Different well-known and meaningful models and their analysis are included on the repository.
Experimental features	Networks generated from systems dynamics models, used for systems dynamics model comparison by complexity.
Data source location	Veszprem, Hungary, University of Pannonia (47.0878073,17.9088153)
Data accessibility	https://doi.org/10.17632/84jw497rwp.1
Related research article	Honti, G., G. Dörgő, and J. Abonyi, *Review and structural analysis of system dynamics models in sustainability science.* Journal of Cleaner Production, 2019.**240**: p. 118015 [1].

**Table:** 

**Value of the Data** • The complexity and the structural patterns of systems dynamics models can be studied by the developed Python program. • The published networks can be used to study how the models of sustainability science are structured. • The networks of state variables can be used as benchmark problems by scientists interested in the network-based analysis of dynamical systems.

## 1 Data

**Fig. 1:**
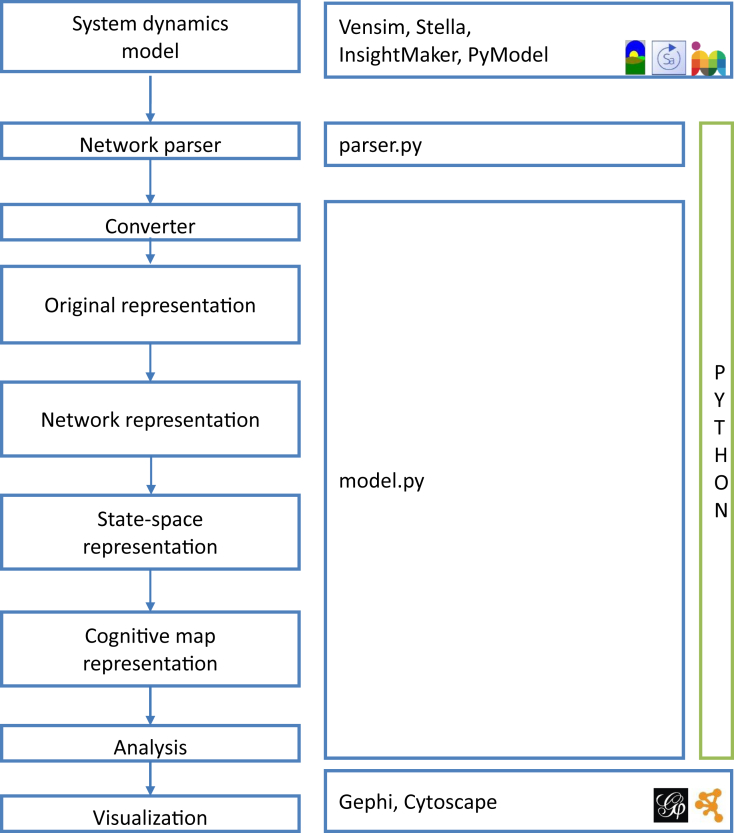
The workflow of the generation of network dataset of systems dynamics models.

The dataset has been generated by the systematic analysis of system dynamics models of sustainability science^1^ , the full analysis is available in the research paper [1]. The included python program extracts networks from models developed in Vensim, Stella, or InsightMaker and processes them according to the workflow shown in Fig. 1. The dataset consists of the following models: the well-known World 3 model [2] and its ascender the World 2 model [3] which use how networks can be extracted from Vensim and InsightMaker, respectively, and some models that are directly defined, including the Wonderland world dynamics model [4], a sustainable development model [5], a water management model [6], a simulation model for water management in Las Vegas [7], the China development model [8], the Urban Dynamics model [9], and a model developed for policy making on recycling in Taiwan [10].

**Fig. 2:**
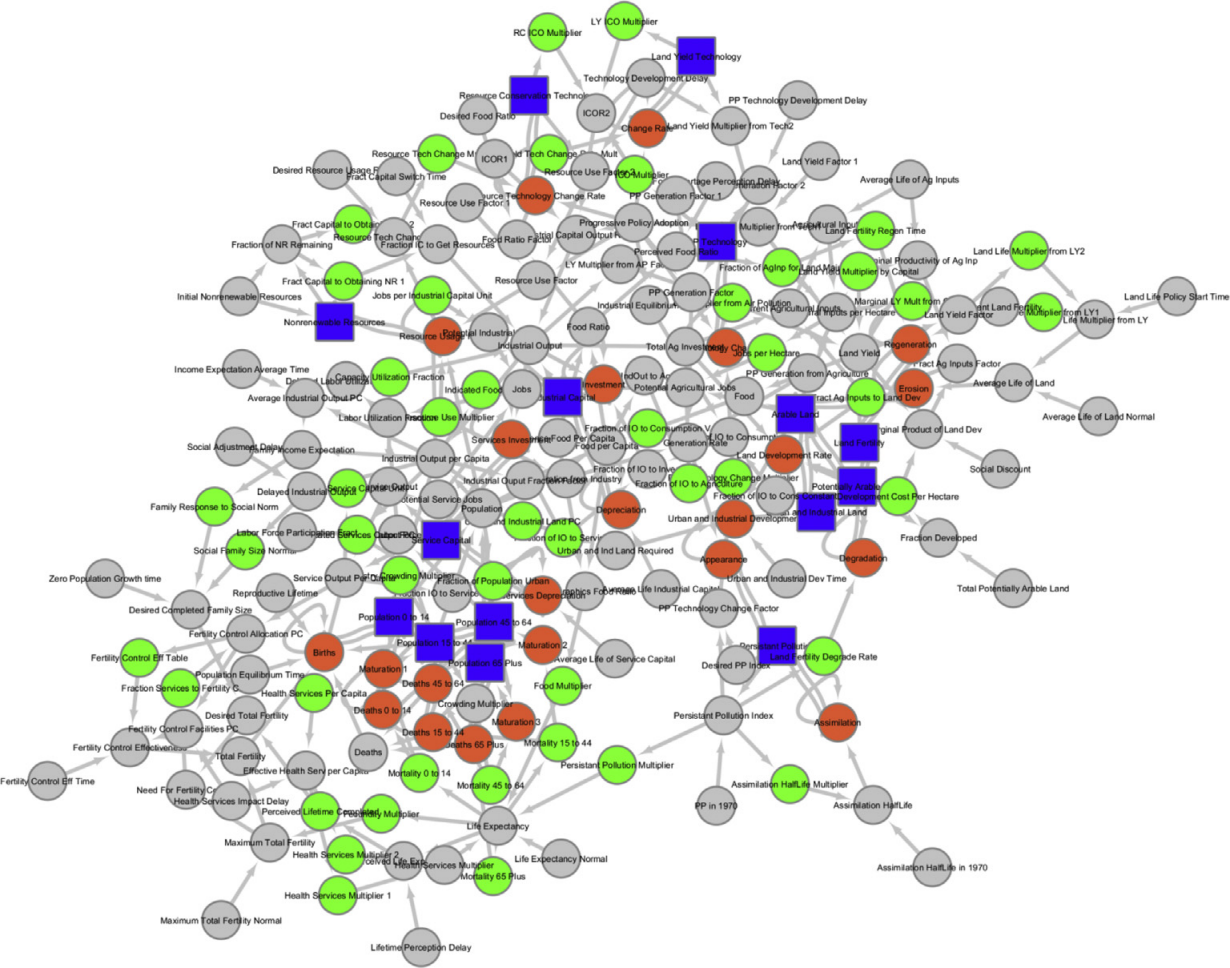
The network of extracted from the World 3 model. The colors of the nodes that represent building elements of the model (blue – state variable, orange – flow, green – variable, grey – parameter).

The dataset covers the transformed networks of state variables, flows and parameters (see Fig. 2 as an example), the networks of state variables, and cognitive maps generated based on the modularity analysis of the state space models. Each representation is evaluated by a set of metrics:

• Number of state variables
• Number of converters
• Number of model parameters
• Number of model connections
• Number of flows
• Number of nodes
• Number of edges
• Diameter of the network
• Density of the networks
• Number of circles
• Number of modules
• Modularity
• Average shortest path
• Average degree
• Wiener index
• Circles:
  ○ $c_{1}$ – Self loops
  ○ $c_{2}$ – Circles with two nodes
  ○ $c_{3}$ – Circles with three nodes
• Triads

## 2 Experimental design, materials, and methods

The software has been developed in Python. The Vensim and Stella systems dynamics models are parsed by an external tool, PySD [11]. InsightMaker models are parsed with the tool that we have developed. Once a model is converted to the PyModel format, it is further processed by extraction of the network of state variables, and generation of a cognitive map, which is the most simplified view of systems dynamics models as the proposed cognitive map representation corresponds to the modules of the networks. The resulted networks are exported as. gexf files, which can be further processed in Gephi or Cytoscape, the most widely used software for network analysis.

## Footnotes

1 Available at: https://doi.org/10.17632/84jw497rwp.1
